# The relationship between dominant follicle development and clinical outcomes of hormone replacement therapy-frozen embryo transfer: a retrospective clinical study

**DOI:** 10.3389/fendo.2023.1192696

**Published:** 2023-06-14

**Authors:** Chenyang Huang, Xiaoyue Shen, Yuan Yan, Huizhi Shan, Qingqing Shi, Jie Mei, Jun Xing

**Affiliations:** ^1^Center for Reproductive Medicine and Obstetrics and Gynecology, Nanjing Drum Tower Hospital, Nanjing University Medical School, Nanjing, China; ^2^Center for Molecular Reproductive Medicine, Nanjing University, Nanjing, China; ^3^Center for Reproductive Medicine and Obstetrics and Gynecology, Drum Tower Clinic Medical College of Nanjing Medical University, Nanjing, China

**Keywords:** hormone replacement therapy, frozen embryo transfer, dominant follicle development, clinical pregnancy rate, live birth rate

## Abstract

**Research question:**

Hormone replacement therapy (HRT) is one of the most used endometrial preparation protocols for frozen embryo transfer (FET) due to the convenience of its administration and stability of pregnancy outcomes. There are several HRT cycles accompanied by the development of dominant follicles. However, the relationship between dominant follicle development and clinical outcomes in HRT-FET cycles remains unclear.

**Design:**

We carried out a retrospective cohort study of 13251 cycles at our reproductive medicine center from 2012 to 2019. Total cycles were divided into two groups according to whether there was dominant follicular development. In addition, we conducted a secondary analysis that used propensity-score matching to reduce confounding variables. A univariate and multivariable logistic regression model was further employed to analyze the effect of dominant follicle development in HRT cycles on clinical pregnancy outcomes.

**Results:**

There was no significant correlation between dominant follicle development in HRT-FET cycles and the clinical pregnancy rate (adjusted OR = 1.162, 95% CI: 0.737-1.832, P = 0.52). In addition, there was a positive correlation between the basic follicle-stimulating hormone (FSH) level and the development of dominant follicles, while there was a negative correlation between antral follicle count (AFC), menstrual cycle length and the development of dominant follicles in HRT cycles.

**Conclusions:**

The development of dominant follicles in HRT-FET cycles does not affect the clinical pregnancy rate, early miscarriage rate and live birth rate. Therefore, it is not necessary to immediately cancel the FET cycle immediately when dominant follicle development is monitored in the HRT-FET cycle.

## Introduction

Due to its safety and comfort, the use of frozen embryo transfer (FET) is becoming increasingly widespread and can maintain satisfactory clinical pregnancy outcomes (1–8). The impact of FET on perinatal outcomes is uncertain, and studies suggesting that FET can bring adverse perinatal outcomes, including hypertension, intrahepatic cholestasis, low birth weight and so on (9–11). However, scholars have also found that FET cycles have better perinatal outcomes than fresh cycle transfers, including lower risks of ectopic pregnancy, preterm birth and placenta previa (12, 13).

Studies have shown that there is no optimal protocol for endometrial preparation during the FET cycle, and the clinical pregnancy rates of various cycle protocols are similar (14–16). Compared with natural cycles or ovulation induction cycles, hormone replacement therapy (HRT) cycle is more controllable, greatly reducing the probability of cycle cancellation (17, 18). Studies have shown that the use of exogenous oestrogen (E_2_) in HRT cycles can inhibit the follicle recruitment. However, 4-16.95% of cycles are still accompanied by the development of dominant follicles (19–21). The increased dosage of exogenous E_2_ or E_2_ administration in advance still does not completely inhibit follicular growth. In addition, the relationship between dominant follicle development occurred in HRT-FET cycles and clinical outcomes remains unclear. Previous studies have suggested that the dominant follicle development or ovulation occurring in HRT-FET cycles leads to an increased serum progesterone (P) level, which makes the timing of endometrial transformation imprecise, and it is recommended that these HRT-FET cycles are cancelled (22). However, such an arrangement will cause patients to miss many FET cycles and delay patients’ time. There is currently no consensus on this problem, and there are few relevant studies(21, 23).

Therefore, we conducted a retrospective analysis of more than 10000 HRT-FET cycles in our reproductive medicine center for the last 7 years. This study aimed to investigate the impact of dominant follicle development on the clinical pregnancy outcomes of HRT-FET cycles. We hope to clarify the appropriate criteria for canceling HRT-FET cycles with dominant follicle development.

## Materials and methods

### Patients

**Figure 1:**
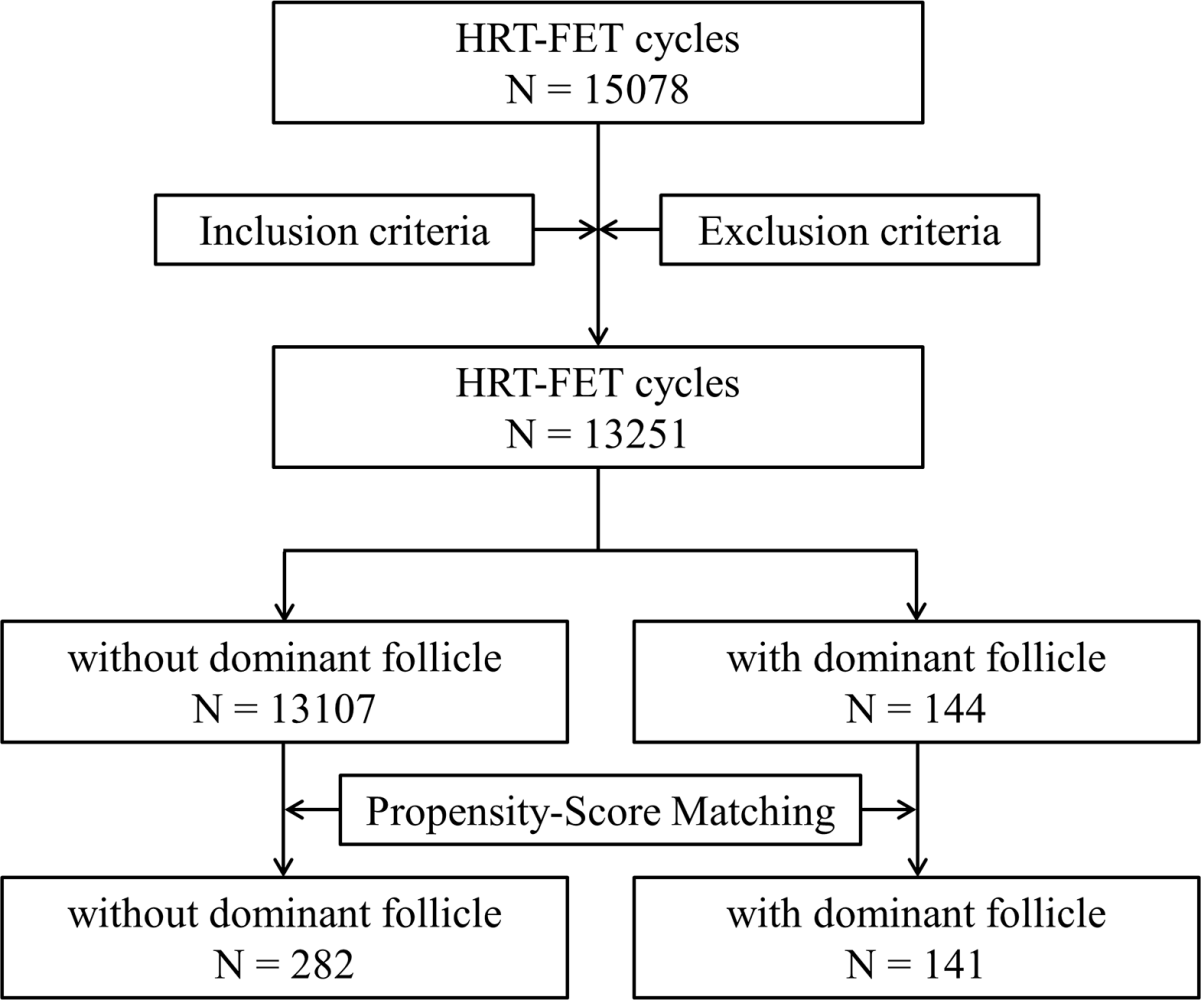
A flow chart of this study.

This retrospective study included FET cycles (not only one cycle per patient) in the reproductive medicine center of Nanjing Drum Tower Hospital from 2012 to 2019 (**Figure 1**). All patients’ endometria were prepared *via* artificial HRT (Femoston, 2 mg estradiol; 2 mg estradiol with 10 mg dydrogesterone, Abbott, USA). Before receiving the HRT-FET cycles, all patients underwent a comprehensive examination to rule out drug and pregnancy contraindications. The exclusion criteria for this study were as follows: 1. Other hormone replacement drugs being used at the same time; 2. Pretreatment with other drugs such as gonadotrophin-releasing hormone agonist (GnRHa); 3. Combined with hydrosalpinx, endometrial lesions or an abnormal uterine environment (uterine fibroids that protrude into the uterine cavity, submucosal fibroids, uterine adhesions, etc.); 4. Moderate or severe endometriosis or adenomyosis. All data were collected using an exclusive internal database of our reproductive medicine center, with patients’ data safeguarded by an advanced threat prevention and periodical password renewals for any user needs. Patients had consented in writing to the use of their anonymized medical records for research purposes. Since both conditions were met, this study had expedited review and approval. The study was approved by the ethics committee of Drum Tower Hospital affiliated with Nanjing University Medical School (No. 2019-217-01).

### Endometrial preparation and thawed embryo transfer

A sex hormone serum test and transvaginal ultrasonography examination were conducted in the early stages of menstruation (the second day of the menstrual cycle). Patients without abnormalities began to orally take a fixed dose of exogenous estradiol for 12-14 days (Femoston, 6 mg estradiol (2 mg t.i.d.)). Serum E_2_, P levels and endometrium thickness were monitored. Patients with a low endometrium thickness would receive additional vaginal medication (Femoston, 2 mg estradiol q.d.). When the endometrium thickness reached a certain standard (≥8 mm), oral estradiol combined with dydrogesterone compound tablets (Femoston, 2 mg estradiol and 10 mg dydrogesterone t.i.d. × 5 or 6 days) were administered with an intramuscular injection of progesterone (progesterone injection, XIANJU PHARMA, Zhejiang, China, 60 mg q.d.) to induce endometrial transformation. Cleavage-stage embryos were thawed and transferred on the fifth day of endometrial transformation and blastocysts were thawed and transferred on the sixth day. Patients usually took Femoston (2 mg estradiol and 10 mg dydrogesterone, t.i.d.) and P sustained-release vaginal gel (Crinone, Merck Serono, Switzerland, 90 mg, q.d.) for luteal support. If an ultrasound indicated that dominant follicle (≥ 14 mm) development was detected in HRT cycles, patients were scheduled to continue monitoring the follicular growth. When the follicle developed to 16-20 mm with appropriate endometrium thickness, human chorionic gonadotropin (hCG, chorionic gonadotrophin injection, Livzon Pharm, China, 10000 IU) was used to induce ovulation and stop the use of exogenous estradiol (Femoston). Patients began to take oral dydrogesterone tablets (Duphaston, Abbott Biologicals B.V., USA, 20 mg b.i.d. × 4 or 6 days) from the second day after hCG administration. Cleavage-stage embryos were thawed and transferred on the fifth day after hCG, and blastocysts were thawed and transferred on the seventh day after hCG. Patients usually took dydrogesterone tablets (Duphaston, 20 mg, b.i.d.) for luteal support. Serum β-human chorionic gonadotropin (β-hCG) was detected 2 weeks after the embryo transfer to determine biochemical pregnancies. The transvaginal ultrasound was examined for patients with an elevated β-hCG level at 4 weeks after the embryo transfer to confirm clinical pregnancies and the number of implanted embryos. A clinical pregnancy was defined as the presence of a gestational sac. Luteal support in pregnant patients was maintained until 2 months after the embryo transfer. Patients were followed up to identify any abnormalities during pregnancy. The spontaneous abortion occurring before 12 weeks of pregnancy is defined as the early miscarriage. The live birth was defined as the delivery of a living newborn after the 28th gestational week and live birth rate was calculated as the ratio of the live birth cycle number to the number of embryo transfer cycles.

### Statistical analysis

The primary outcome of our study was the clinical pregnancy rate which was defined as the ratio of number of patients with the clinical pregnancy to the number of patients who received an embryo transfer. The live birth rate and early miscarriage rate was considered as a secondary outcome in this study. All cycles were divided into groups A and B according to whether there was dominant follicular development. During the FET cycles, the relationship between dominant follicle development and clinical pregnancy rate was evaluated. In addition, we used propensity-score methods to reduce confounding variables (female age, male age, BMI, baseline FSH, AFC, Length of menstrual cycle, infertility duration, type of infertility, number of previous transfer cycles). Matching was performed with the use of a 1:2 matching protocol without a replacement, with a caliper width equal to 0.01 of the standard deviation of the propensity score logit. Univariate analysis was used to preliminarily evaluate variables related to the clinical pregnancy outcome, and a multivariable logistic regression model was further employed to analyze the effect of dominant follicle development in HRT cycles on the clinical pregnancy rate. The parameters were explained as Mean ± Standard Deviation (SD). All analyses were performed with R (http://www.R-project.org) and EmpowerStats software (www.empowerstats.com, X&Y solutions, Inc. Boston MA). A p value < 0.05 was considered statistically significant.

### Ethics

This study has received ethical approval from the ethics committee of Nanjing Drum Tower Hospital.

## Results

### Characteristics of all HRT-FET cycles

This study involved 13251 HRT-FET cycles. The prevalence of dominant follicle occurrence in retrospectively evaluated HRT-FET cycles was 1.08%. In 13107 cycles (group A), there was no dominant follicle development in HRT cycles, while in 144 cycles (group B), there was dominant follicle development. The infertility duration of these two groups was similar. The female age, basal serum follicle-stimulating hormone (FSH) level, number of previous embryo transfers and endometrium thicknesses of group A were lower than those of group B, but the body mass index (BMI), antral follicle count (AFC) and menstrual cycle length of group A were greater than those of group B. There was no significant difference between the two groups in the number of transferred embryos, the proportion of transferred blastocysts, clinical pregnancy rate, early miscarriage rate and live birth rate (**Table 1**).

**Table 1:** Characteristics of HRT-FET cycles with or without dominant follicle development.

	HRT without dominant follicle development (n=13107)	HRT with dominant follicle development (n=144)	P value
**Female age, years**	31.7 ± 5.3	34.2 ± 5.7	<0.001
**Male age, years**	33.2 ± 6.0	35.8 ± 6.8	<0.001
**BMI, kg/m^2^**	22.6 ± 3.2	22.1 ± 2.8	0.13
**Baseline FSH, IU/L**	7.7 ± 3.4	9.2 ± 4.8	<0.001
**AFC, n**	15.8 ± 6.6	11.0 ± 6.1	<0.001
**Length of menstrual cycle, days**	43.4 ± 38.1	30.1 ± 9.0	<0.001
**Infertility duration, years**	4.0 ± 3.2	4.1 ± 3.4	0.67
**Type of infertility**			0.01
**Primary infertility, n** **Secondary infertility, n**	6725 (51.3%) 6382 (48.7%)	58 (40.3%) 86 (59.7%)	
**Transfer cycles, n**	1.8 ± 1.5	2.2 ± 2.0	0.02
**Endometrial thickness, mm**	9.3 ± 1.4	10.0 ± 1.8	<0.001
**Number of embryos, n**	1.6 ± 0.5	1.6 ± 0.5	0.09
**Different types of embryos transferred**			0.16
**Cleavage-stage embryos, n**	7624 (58.2%)	97 (67.4%)	
**Blastocyst, n**	5483 (41.8%)	47 (32.6%)	
**Clinical pregnancy rate**	56.5% (7406)	51.4% (74)	0.21
**Clinical pregnancy rate** **of cleavage-stage embryos**	49.2% (3750)	47.4% (46)	0.73
**Clinical pregnancy rate** **of blastocysts**	66.7% (3656)	59.6% (28)	0.30
**Early miscarriage rate**	12.3% (914)	14.9% (11)	0.51
**Live birth rate**	46.7% (6126)	41.0% (59)	0.17

### Outcomes based on multivariate regression analysis

To control confounding variables, a multivariable logistic regression model was used. A preliminary univariate analysis was used to identify confounding factors that might affect clinical pregnancy outcomes (**Table S1**). Baseline factors (female age, menstrual cycle length, BMI, AFC, baseline FSH level, infertility type, infertility duration and number of previous embryo transfers) and treatment factors (number of transferred embryos, type of transferred embryos and endometrium thickness) were selected as the adjustment variables of the multivariate regression analysis. There was no significant correlation between dominant follicle development in HRT-FET cycles and clinical pregnancy rate (**Table S2**, adjusted OR = 1.133, 95% CI: 0.797-1.609, P = 0.49).

### Comparisons after the propensity-score matching

Considering the differences in the number of cycles and baseline characteristics between the two groups (**Table 1**), we adopted propensity-score matching to identify the cycle cohort with similar baseline characteristics. As shown in **Table 2**, after propensity-score matching, the baseline characteristics of patients in the two groups were similar. Endometrium thickness in group A was lower than that in group B, but there was no difference in the number of transferred embryos and the proportion of transferred blastocysts between the two groups (**Table 2**). There was no significant difference in the clinical pregnancy rate, early miscarriage rate and live birth rate between the two groups (**Table 2**). In addition, univariate analysis was also used to identify confounding factors that may affect clinical pregnancy outcomes (**Table S3**). Baseline factors (female age, male age, AFC, baseline FSH level, infertility duration and number of previous embryo transfer cycles) and treatment factors (endometrium thickness, number of transferred embryos and type of transferred embryos) were selected as the adjustment variables of the multivariate regression analysis. There was no significant correlation between dominant follicular development in HRT-FET cycles and clinical pregnancy rate after propensity-score matching (**Table S4**, adjusted OR = 1.162, 95% CI: 0.737-1.832, P = 0.52).

**Table 2:** Characteristics of HRT-FET cycles with or without dominant follicle development after Propensity-Score Matching.

Variable	Group A: without dominant follicle development (n=282)	Group B: with dominant follicle development (n=141)	P value
**Female age, years**	34.4 ± 5.8	34.1 ± 5.6	0.61
**Male age, years**	35.8 ± 6.7	35.7 ± 6.7	0.87
**BMI, kg/m^2^**	22.2 ± 2.8	22.1 ± 2.8	0.64
**Baseline FSH, IU/L**	9.0 ± 4.2	8.9 ± 4.1	0.84
**AFC, n**	11.3 ± 6.3	11.2 ± 6.0	0.91
**Length of menstrual cycle, days**	30.0 ± 6.4	30.2 ± 9.1	0.85
**Infertility duration, years**	4.5 ± 3.9	4.1 ± 3.4	0.31
**Type of infertility**			0.86
**Primary infertility, n** **Secondary infertility, n**	126 (42.6%) 162 (57.4%)	58 (41.1%) 83 (58.9%)	
**Transfer cycles, n**	2.2 ± 1.8	2.1 ± 1.7	0.48
**Endometrial thickness, mm**	9.2 ± 1.4	10.0 ± 1.9	<0.001
**Number of embryos, n**	1.6 ± 0.5	1.6 ± 0.5	0.40
**Different types of embryos transferred**			0.46
**Cleavage-stage embryos, n**	200 (70.9%)	94 (66.7%)	
**Blastocyst, n**	82 (29.1%)	47 (33.3%)	
**Clinical pregnancy rate**	45.7% (129)	51.8% (73)	0.29
**Early miscarriage rate**	10.9% (14)	15.1% (11)	0.38
**Live birth rate**	37.9% (107)	41.8% (59)	0.44

### Correlation between characteristics and the occurrence of dominant follicular development

Univariate analysis showed that female age, BMI, basal FSH level, AFC, menstrual cycle length and the type of infertility of patients was related to the development of dominant follicles in HRT-FET cycles (**Table 3**). Similarly, propensity score matching was conducted for the infertility duration. The results of univariate analysis after propensity score matching showed that there was a positive correlation between the basic FSH level and the development of dominant follicles in HRT-FET cycles, while there was a negative correlation between AFC, menstrual cycle length and the development of dominant follicles in HRT-FET cycles (**Table S5**). Dominant follicle development is more likely to be detected in patients of an older age and with a higher basal FSH level, shorter menstrual cycle, and less AFC.

**Table 3:** Univariate analysis of dominant follicle development in total HRT-FET cycles.

Total cycles	Adjusted OR	95% CI	p value
**Female age**	1.802	1.052-1.113	<0.001
**BMI**	0.945	0.895-0.998	0.04
**Baseline FSH, IU/L**	1.075	1.046-1.105	<0.001
**AFC**	0.895	0.872-0.920	<0.001
**Length of menstrual cycle, days**	0.935	0.907-0.964	<0.001
**Infertility duration, years**	1.005	0.955-1.057	0.85
**Type of infertility**			0.008
**Primary infertility, n** **Secondary infertility, n**	1.000 1.562	1.000 1.182-2.184	

## Discussion

About half of the embryos have been cryopreserved in Europe and the United States (24, 25) and FET can help carry out a selective single embryo transfer strategy and reduce the probability of multiple pregnancies (25–28). In artificial HRT cycles, we do not need to consider whether the patient’s menstruation is regular or not and can flexibly arrange the time of embryo transfer, leading to the wide use of HRT cycles (29, 30). GnRHa pretreatment has been believed to be able to inhibit abnormal LH levels caused by exogenous E_2_ and improve endometrial function in the HRT cycles while without significantly improvement on the clinical pregnancy outcomes of the HRT-FET cycle (31–33). At present, HRT cycles without pituitary suppression are widely used, and most cycles are cancelled because of dominant follicle development, which increases the unnecessary burden on patients (22). Nevertheless, dominant follicle development cannot be completely avoided in HRT-FET cycles. The prevalence of dominant follicle occurrence in retrospectively evaluated HRT-FET cycles was 1.08%. Therefore, the question of how to deal with dominant follicle development in HRT-FET cycles needs to be solved urgently.

The effect of follicular growth in HRT-FET cycles on clinical outcomes was investigated in our study. In the HRT-FET cycles without pituitary down-regulation, dominant follicular development had no significant effect on the clinical pregnancy rate, early miscarriage rate and live birth rate. After propensity score matching, the clinical pregnancy rate in the group with follicular growth was similar with that in the conventional HRT-FET group. In the past, few studies explored the impact of follicular development or ovulation in HRT-FET cycles on clinical pregnancy outcomes. Similar results were obtained in a retrospective study in 2013, which suggested that the clinical pregnancy rate of the dominant follicle development group was a little higher than that of the group without follicle development without statistical significance, and the miscarriage rate was lower significantly (21). This study suggested that follicular development produces endogenous E_2_, which acts in synergy with exogenous E_2_ to accelerate endometrial growth. Moreover, due to the insufficient development of granulosa cells in follicles needed to ovulate or luteinize, the level of endogenous P did not significantly increase, which would not affect the endometrial transformation time of adding exogenous P (21). Although our results are similar, we took more detailed monitor of follicles during the HRT cycle accompanying the development of dominant follicles and arranged the appropriate timing of endometrial transformation and embryo transfer according to the follicle development. In addition, the inclusion time of this previous study was short, and the number of FET cycles was small. Our study further expanded the time span and number of FET cycles involved. The patients in our study received different types of embryo transfers. Variables affecting clinical outcomes, such as type of transferred embryos, were included in the multivariate regression analysis. In our reproductive medicine center, when ovulation was found at the first ultrasound and sex hormone measurement, the FET cycle was directly cancelled. Therefore, we lack the HRT-FET cycles with preovulation compared with the previous study.

Our study confirmed that follicular development in HRT-FET cycles did not affect the clinical pregnancy outcome. For patients who had ovulated by the time of the first monitoring, the transfer cycle was directly cancelled. Therefore, whether these patients with dominant follicular development in HRT-FET cycles have similar clinical characteristics is worthy of further exploration. For patients with a high probability of dominant follicular development in HRT-FET cycles, the time of their first serum sex hormone and ultrasound examination can be appropriately advanced to put their follicular development in a controllable range and avoid unnecessary cycle cancellation. Therefore, we analyzed the correlation between clinical baseline characteristics and the occurrence of dominant follicular development. The results showed that there was a positive correlation between the basic FSH level and the development of dominant follicles in HRT-FET cycles, while there was a negative correlation between AFC, menstrual cycle length and the development of dominant follicles in HRT-FET cycles (**Table S5**). Dominant follicle development is more likely to be detected in patients of an older age and with a higher basal FSH level, shorter menstrual cycle and less AFC. Previous studies have confirmed that for elderly patients with decreased ovarian reserve function and less AFC, the secretion of inhibin is reduced and the inhibition of FSH is limited. Therefore, their follicle development occurs relatively easily in advance (34). Combined with our study, for such older patients with decreased ovarian reserve function, the application of exogenous E_2_ in the early menstrual period may occasionally fail to inhibit follicular recruitment and development. However, studies suggest that HRT is a more appropriate choice for specific patients, such as women of an increased age (> 40 years old), significantly decreased ovarian function and shortened menstrual cycle (19, 35). Therefore, for such patients, we need to pay more attention to the follicular development in their HRT-FET cycles. We can advance the time of their first return to hospital for examination as appropriate, to avoid unnecessary cancellation of the FET cycle.

There are still some shortcomings in current study. Our study only includes the HRT-FET cycle of Femoston and does not include other exogenous E_2_ or other pre-treatments. Due to the limitations of the data system, our study lacks several baseline characteristics for the patients. For patients who experience preovulation in HRT cycles, we usually cancel the FET cycle, and this study did not include this group of patients. In addition, the long-term follow-up for 7 years is another limitation of our study. Of course, the main limitation of this study lies in the retrospective design. Therefore, in a real-life setting, these results are cautiously applicable.

## Conclusions

In conclusion, our retrospective study suggests that the development of dominant follicles in HRT-FET cycles does not affect the clinical pregnancy rate, early miscarriage rate and live birth rate. Therefore, it is not necessary to immediately cancel the FET cycle when dominant follicle development is monitored in the HRT-FET cycle.

## Data availability statement

The raw data supporting the conclusions of this article will be made available by the authors, without undue reservation.

## Author contributions

CH, JM and JX contributed to study design, execution, acquisition, analysis, and interpretation of data, manuscript drafting, and critical discussion. XS, YY, HS and QS contributed to acquisition and interpretation of data, manuscript drafting, and critical discussion. All authors contributed to the article and approved the submitted version.
