# Supplementary material for: The relationship between dominant follicle development and clinical outcomes of hormone replacement therapy-frozen embryo transfer: a retrospective clinical study
Source: Front Endocrinol (Lausanne). 2023 Jun 14;14:1192696. doi: 10.3389/fendo.2023.1192696 (PMC10306306; doi:10.3389/fendo.2023.1192696)
Supplement: Supplementary file 1 [file Table_1.docx]

**Table S1.** Univariate analysis of total HRT-FET cycles.

| **Total cycles** | **Adjusted OR** | **95% CI** | **p value** |
| --- | --- | --- | --- |
| **Female age** | 0.899 | 0.892-0.905 | <0.001 |
| **Male age** | 0.923 | 0.918-0.929 | <0.001 |
| **BMI** | 0.986 | 0.975-0.997 | 0.01 |
| **Baseline FSH, IU/L** | 0.926 | 0.916-0.937 | <0.001 |
| **AFC** | 1.071 | 1.065-1.077 | <0.001 |
| **Length of menstrual cycle, days** | 1.005 | 1.004-1.006 | <0.001 |
| **Infertility duration, years** | 0.933 | 0.923-0.944 | <0.001 |
| **Type of infertility** | | | <0.001 |
| **Primary infertility, n**  **Secondary infertility, n** | 1.000  0.669 | 1.000  0.624-0.717 |  |
| **Transfer cycles, n** | 0.775 | 0.754-0.796 | <0.001 |
| **Endometrial thickness, mm** | 1.095 | 1.067-1.123 | <0.001 |
| **Number of embryos, n** | 1.950 | 1.815-2.096 | <0.001 |
| **Different types of embryos transferred** | | | <0.001 |
| **Cleavage-stage embryos, n**  **Blastocyst, n** | 1  2.063 | 1  1.921-2.216 |  |
| **HRT cycles** | | | 0.22 |
| **without dominant follicle development**  **with dominant follicle development** | 1  0.814 | 1  0.586-1.130 |  |
