# Supplementary material for: The relationship between dominant follicle development and clinical outcomes of hormone replacement therapy-frozen embryo transfer: a retrospective clinical study
Source: Front Endocrinol (Lausanne). 2023 Jun 14;14:1192696. doi: 10.3389/fendo.2023.1192696 (PMC10306306; doi:10.3389/fendo.2023.1192696)
Supplement: Supplementary file 2 [file Table_2.docx]

**Table S2.** Multivariate analysis for dominant follicle development in HRT-FET cycles involved in the clinical pregnancy rate.

| **Variable** | **Adjusted OR** | **95% CI** | | **p value** | |
| --- | --- | --- | --- | --- | --- |
| **dominant follicle development in HRT-FET cycles** | 1.133 | | 0.797-1.609 | | 0.49 |

**Adjust for**: female age, menstrual cycle length, BMI, AFC, baseline FSH level, infertility type, infertility duration, number of previous embryo transfer cycles, number of transferred embryos, type of transferred embryos and endometrial thickness.
