# Supplementary material for: The relationship between dominant follicle development and clinical outcomes of hormone replacement therapy-frozen embryo transfer: a retrospective clinical study
Source: Front Endocrinol (Lausanne). 2023 Jun 14;14:1192696. doi: 10.3389/fendo.2023.1192696 (PMC10306306; doi:10.3389/fendo.2023.1192696)
Supplement: Supplementary file 3 [file Table_3.docx]

**Table S3.** Univariate analysis of total HRT-FET cycles after Propensity-Score Matching.

| **Total cycles** | **Adjusted OR** | **95% CI** | **p value** |
| --- | --- | --- | --- |
| **Female age** | 0.887 | 0.854-0.921 | <0.001 |
| **Male age** | 0.931 | 0.903-0.961 | <0.001 |
| **BMI** | 1.004 | 0.938-1.076 | 0.90 |
| **Baseline FSH, IU/L** | 0.906 | 0.860-0.956 | <0.001 |
| **AFC** | 1.078 | 1.043-1.114 | <0.001 |
| **Length of menstrual cycle, days** | 1.024 | 0.995-1.054 | 0.11 |
| **Infertility duration, years** | 0.949 | 0.901-1.000 | 0.04 |
| **Type of infertility** | | | 0.12 |
| **Primary infertility, n**  **Secondary infertility, n** | 1.000  0.733 | 1.000  0.497-1.079 |  |
| **Transfer cycles, n** | 0.794 | 0.701-0.900 | <0.001 |
| **Endometrial thickness, mm** | 1.095 | 1.067-1.123 | <0.001 |
| **Number of embryos, n** | 1.917 | 1.289-2.851 | 0.001 |
| **Different types of embryos transferred** | | | 0.005 |
| **Cleavage-stage embryos, n**  **Blastocyst, n** | 1  1.826 | 1  1.201-2.777 |  |
| **HRT cycles** | | | 0.24 |
| **without dominant follicle development**  **with dominant follicle development** | 1  1.273 | 1  0.849-1.909 |  |
