# Supplementary material for: The relationship between dominant follicle development and clinical outcomes of hormone replacement therapy-frozen embryo transfer: a retrospective clinical study
Source: Front Endocrinol (Lausanne). 2023 Jun 14;14:1192696. doi: 10.3389/fendo.2023.1192696 (PMC10306306; doi:10.3389/fendo.2023.1192696)
Supplement: Supplementary file 4 [file Table_4.docx]

**Table S4.** Multivariate analysis for dominant follicle development involved in the clinical pregnancy rate after Propensity-Score Matching.

| **Variable** | **Adjusted OR** | **95% CI** | | **p value** | |
| --- | --- | --- | --- | --- | --- |
| **dominant follicle development in HRT-FET cycles** | 1.162 | | 0.737-1.832 | | 0.52 |

**Adjust for**: female age, male age, AFC, baseline FSH level, infertility duration, number of previous embryo transfer cycles, endometrial thickness, number of transferred embryos and type of transferred embryos.
