# Supplementary material for: The relationship between dominant follicle development and clinical outcomes of hormone replacement therapy-frozen embryo transfer: a retrospective clinical study
Source: Front Endocrinol (Lausanne). 2023 Jun 14;14:1192696. doi: 10.3389/fendo.2023.1192696 (PMC10306306; doi:10.3389/fendo.2023.1192696)
Supplement: Supplementary file 5 [file Table_5.docx]

**Table S5.** Univariate analysis of dominant follicle development in total HRT-FET cycles after Propensity-Score Matching.

| **Total cycles** | **Adjusted OR** | **95% CI** | **p value** |
| --- | --- | --- | --- |
| **Female age** | 1.018 | 0.983-1.054 | 0.32 |
| **BMI** | 0.933 | 0.867-1.003 | 0.06 |
| **Baseline FSH, IU/L** | 1.044 | 1.003-1.086 | 0.04 |
| **AFC** | 0.928 | 0.898-0.958 | <0.001 |
| **Length of menstrual cycle, days** | 0.951 | 0.926-0.978 | <0.001 |
| **Infertility duration, years** | 0.840 | 0.787-1.007 | 0.09 |
| **Type of infertility** | | | 0.07 |
| **Primary infertility, n**  **Secondary infertility, n** | 1.000  1.546 | 1.000  1.031-2.318 |  |
